# Protective Effect of γ-mangostin Isolated from the Peel of *Garcinia mangostana* against Glutamate-Induced Cytotoxicity in HT22 Hippocampal Neuronal Cells

**DOI:** 10.3390/biom11020170

**Published:** 2021-01-27

**Authors:** Ji Yun Baek, Kiwon Jung, Young-Mi Kim, Hyun-Young Kim, Ki Sung Kang, Young-Won Chin

**Affiliations:** 1 Department of Preventive Medicine, College of Korean Medicine, Gachon University, Seongnam 13120, Korea; wldbsttn@naver.com; 2 Department of Food Science, Gyeongnam National University of Science and Technology, Jinju 52725, Korea; 3 Institute of Pharmaceutical Sciences, College of Pharmacy, CHA University, Sungnam 13844, Korea; pharmj@cha.ac.kr; 4 College of Pharmacy and Research Institute of Pharmaceutical Sciences, Seoul National University, 1 Gwanak-lo, Gwanak-gu, Seoul 08826, Korea; 0210121@hanmail.net

**Keywords:** HT22 cells, glutamate, γ-mangostin, oxidative stress, apoptosis

## Abstract

The aim of the present study was to examine the protective effect of γ-mangostin, a component of the mangosteen shell, against oxidative damage to nerve cells induced by excessive glutamate, a known excitatory neurotransmitter. To investigate the effect of γ-mangostin on apoptosis, 5 mM of glutamate was used to induce apoptotic cell death in mouse hippocampal HT22 cells. In this study, γ-mangostin was found to exert a stronger protection than N-acetyl cysteine against glutamate-induced cell damage. γ-Mangostin showed prevented glutamate-induced apoptosis in HT22 cells by reducing the production of reactive oxygen species and stimulating the expression of heme oxygenase-1 protein. In addition, glutamate significantly induced the accumulation of intracellular calcium ions, whereas treatment with γ-mangostin markedly reduced it. Hoechst 33342 staining showed an improvement in glutamate-induced nuclear condensation following γ-mangostin treatment. Furthermore, the number of annexin V-positive cells was significantly reduced following treatment with γ-mangostin. Western blot analysis showed the inhibition of glutamate-induced mitogen-activated protein kinase phosphorylation by γ-mangostin. γ-mangostin also inhibited the regulation of the intrinsic mitochondrial apoptotic pathway. Thus, the results of this study suggest that γ-mangostin is an active ingredient of mangosteen and exerts neuroprotective activities in HT22 cells.

## 1. Introduction

There has been a growing interest in the prevention of neurodegenerative diseases owing to the steady increase in the elderly population. Neurodegenerative diseases typically include Alzheimer’s disease, Parkinson’s disease, dementia, and frontal lobe degeneration, among others [1]. Neuronal cell death is a causative factor of neurodegenerative disease [2,3], and in vitro and in vivo studies have contributed to our understanding of the mechanism of neuronal cell death [4].

Glutamate works as synaptic plasticity that is capable of continuous changes in structure and function depending on the degree of activity in the brain, which is related to cognitive functions such as learning and memory [5]. However, if it is over-activated, it causes epilepsy and excitotoxicity as it acts as a neurotoxin [6]. As a result, the cell firing threshold gets abnormally lowered, damaging apoptosis, oxidative stress, and brain cells, which are reported as the main factors to cause brain diseases [7]. Since HT22 cells do not have ionotropic glutamate receptors, it is possible to exclude the excitotoxic agent caused by glutamate that causes cell death [8]. It is known that active oxygen species excessively generated by glutamate in HT22 cells promote apoptosis gene expression by activating the mitogen-activated protein kinase (MAPK) signaling pathway caused by the apoptosis signal in order to induce apoptosis [9]. Furthermore, the mitochondria-related apoptosis mechanism is mainly caused by changes in the expression of various proteins that act as apoptotic regulators, which is related to a decrease in bcl-2 of the antiapoptotic protein and an increase in bax of the proapoptotic protein [10].

Interventions aimed at preventing neuronal cell death using therapeutic agents may alleviate its progression, but there are still no specific treatment methods for the condition. Furthermore, the treatment of neurological disorders with currently known drugs is often accompanied by adverse side effects. Therefore, to overcome these challenges, there has been an increasing interest in identifying novel protective agents derived from natural products.

It is reported that natural products are effective in preventing neuronal cell death and recovering cognitive ability in vitro and in animal models of neurological orders [11]. In addition, certain natural products have been shown to have various antioxidant, anti-inflammatory, and antiapoptotic activities that provide beneficial effects against neuronal cell death. Therefore, identifying natural products that inhibit neuronal cell death is important for suppressing the side effects of currently known drugs and controlling neurological disorders.

Mangosteen (*Garcinia mangostana*) is an edible fruit native to Malaysia, but it is also grown in other south Asian countries such as in Indonesia, Taiwan, the Philippines, and India. The pigment of mangosteen flesh contains tannins and is used as a medicine to treat skin infections and wounds, to relieve muscle and bone pain, and in eating disorders.

Previous studies have demonstrated that mangosteen peel contains various bioactive components, including α-, β-, and γ-mangostin, that exhibit pharmacological activities such as antioxidant, anti-inflammatory, antibacterial, and antidiabetic effects. Among these components, γ-mangostin is known for its efficacy as an anti-inflammatory and antiallergic agent [12,13,14]. However, the protective effect of γ-mangostin on HT22 cells has not yet been confirmed. We investigated the neuroprotective activity and molecular mechanism of γ-mangostin on HT22 cells. Therefore, in this study, we investigated the neuroprotective activity and molecular mechanisms of γ-mangostin in brain cells.

## 2. Materials and Methods

### 2.1. Cell Culture

Cells of the HT22, the immortalized mouse hippocampal cell line, derived from the mouse hippocampus, were incubated in Dulbecco’s modified Eagle’s medium (DMEM; Corning, Manassas, VA, USA) supplemented with 100 U/mL penicillin, 100 μg/mL streptomycin, and 10% fetal bovine serum (FBS, Gibco, Grand Island, NY, USA). The cells were maintained at 37 °C in a 5% CO_2_ incubator.

### 2.2. Measurement of Cell Viability

HT22 cells were seeded at a density of 1 × 10^4^ cells/well in a 96-well plate and incubated for 24 h to allow for cell attachment. The HT22 cell death was measured using a cell viability assay kit (EZ-Cytox; Daeil Lab Service, Seoul, Korea), according to the manufacturer’s instructions. Cells were treated with glutamate (Sigma, St. Louis, MO, USA), γ-mangostin (prepared as described in Supplementary Materials), and positive control N-acetyl cysteine (Sigma, St. Louis, MO, USA) at each concentration for 24 h, and 10 ul of EZ-cytox reagent were added. Absorbance at 450 nm was measured using an E-Max microplate reader (Molecular Devices, Sunnyvale, CA, USA). The cell viability was calculated as the percentage of viable cells over control cells. All morphological changes were confirmed using an IX50 fluorescent microscope (Olympus, Tokyo, Japan) equipped with a CCD camera.

### 2.3. Measurement of Reactive Oxygen Species (ROS)

To determine the intracellular ROS levels, cells were treated with 2’,7’-dichlorodihydrofluorescein diacetate (H2DCFDA; Sigma, St. Louis, MO, USA), and fluorescence was measured using a fluorescent plate reader (SPARK 10M; Tecan, Männedorf, Switzerland) [15]. HT22 cells were plated at a density of 1 × 10^4^ cells in 96-well black plates and incubated for 24 h. After treatment with different concentrations of γ-mangostin and 5 mM glutamate for 8 h, 10 μM H2DCFDA was added to the wells, and the plates were incubated for 30 min. Residual H2DCFDA that did not bind to ROS was removed using PBS, and the fluorescence intensity of DCF at 495 nm/517 nm (ex/em) was measured. Fluorescent images were obtained using a fluorescence microscope (Olympus, Tokyo, Japan) equipped with a CCD camera.

### 2.4. Measurement of Intracellular Calcium

To determine the intracellular Ca^2+^ levels, 2 × 10^5^ cells were plated in six-well plates [16]. The cells were then treated with 5 mM glutamate and/or γ-mangostin for 8 h. The cells were incubated with 2.5 μM Fluo-4 AM (Invitrogen, Eugene, OR, USA) solution dissolved in DMEM for 30 min at 37 °C. The medium was removed from each well, and the cells were washed twice with PBS. Fluorescent images were obtained with a fluorescence microscope equipped with a CCD camera (Olympus). Quantification of Ca^2+^ was conducted using Image J software (Version 1.51J; National Institute of Health, Bethesda, MD, USA).

### 2.5. Nuclear Staining

To assess the morphological changes in the nucleus, cells were plated at a density of 2 × 10^5^ cells/well in six-well plates and incubated for 24 h. γ-Mangostin was treated with 5 mM glutamate and incubated for 12 h, and was then stained for 10 min using a 10 μM Hoechst 33342 (Sigma, St. Louis, MO, USA). The morphology of the nucleus was visualized using a fluorescence microscope (Olympus, Tokyo, Japan) equipped with a CCD camera.

### 2.6. Measurement of Apoptotic Cell Death Using Tali-Image-Based Cytometry

To assess the effect of γ-mangostin on apoptotic cell death, 2 × 10^5^ cells were plated in a six-well plate and incubated for 24 h [17]. Cells were treated with 5 mM glutamate or γ-mangostin for 12 h, harvested, and washed with PBS. Cells were then treated with annexin-binding buffer and annexin V Alexa Fluor 488 (Invitrogen, Eugene, OR, USA) for 20 min in the dark. The cells were stained with propidium iodide in annexin-binding buffer for 5 min. After removal of the residual fluorescent material using PBS, a Tail-Image-based cytometer was used to measure the percentage of apoptotic cells using the TaliPCApp (version 1.0) program (Invitrogen, Eugene, OR, USA). The results are presented as the percentage of cells stained with annexin V divided by the total cell number in each group.

### 2.7. Western Blot Analysis

To perform the western blot analysis, 2 × 10^5^ cells were plated in six-well plates and treated with 5 mM glutamate or γ-mangostin [18]. The harvested cells were lysed with RIPA buffer containing a protease inhibitor cocktail (Roche, Indianapolis, IN, USA). The isolated protein fraction was separated using 10% sodium dodecyl sulfate-polyacrylamide gel electrophoresis (SDS-PAGE) and then transferred onto nitrocellulose membranes (Merck Millipore, Darmstadt, Germany). The membranes were blocked with 5% skim milk overnight, probed with primary antibodies against heme oxygenase-1 (HO-1), phospho-JNK (p-JNK), JNK, phospho-p38 (p-p38), p38, Bcl-2, Bax, and glyceraldehyde 3-phosphate dehydrogenase (GAPDH) for 2 h, and treated with suitable secondary antibodies as required (Cell Signaling Technology, New England Biolabs, Ipswich, MA, USA). For the imaging of the immunoreactive bands, SuperSignal West Femto Maximum Sensitivity Substrate (Thermo Scientific, Rockford, IL, USA) was used, and the protein bands were visualized using a Fusion Solo Chemiluminescence System (PEQLAB Biotechnologie GmbH, Erlangen, Germany).

### 2.8. Statistical Analysis

All experiments were repeated more than three times, and the data are presented as the mean ± standard error of the mean (SEM). We used the GraphPad PRISM statistical package (ver 5.00, Graphpad software inc., San Diego, USA) and conducted a one-way ANOVA at *p* < 0.05.

## 3. Results

**Figure 1:**
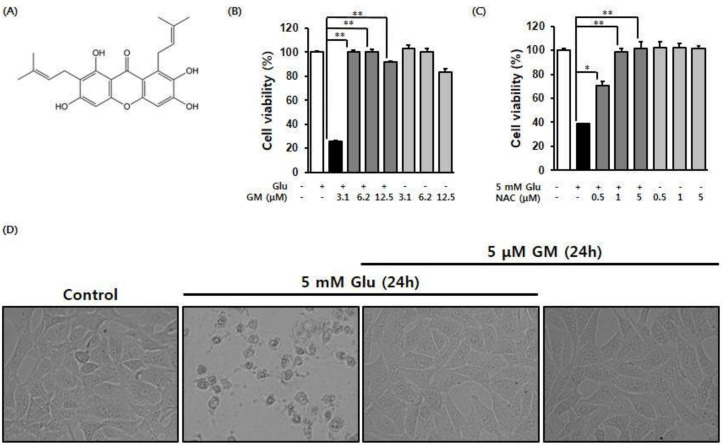
Protective effect of γ-mangostin (GM) and N-acetyl cysteine against (NAC) on glutamate-induced cell death in HT22 cells. (**A**) Chemical structure of GM isolated from mangosteen. (**B**) Effect of GM on glutamate-induced cell death in HT22 cells (mean ± S.E.M. * *p* < 0.05 ** *p* < 0.01 versus glutamate-treated HT22 cells). (**C**) Effect of NAC on glutamate-induced cell death in HT22 cells. Bars represent the percentage of viable cells. Data are expressed as the mean ± standard deviation (SD). ** *p* < 0.01 versus the Glu-treated cells. (**D**) After exposure to 5 mM Glu with/without GM for 24 h; cell images were obtained using a microscope (20× magnification).

The effect of γ-mangostin on glutamate-induced HT22 cell death was tested using cell viability assays (Figure 1A). To determine the effect of γ-mangostin on HT22 neuronal cell apoptosis induced by 5 mM glutamate, HT22 cells in culture were treated with various concentrations of γ-mangostin (3.1, 6.2, and 12.5 μM) for 24 h. Compared to the glutamate-treated group of 25.76 ± 0.43%, the cell viability that was reduced by glutamate was significantly increased to 101.28 ± 1.71, 100 ± 1.35, and 91.68 ± 1.02% in the group treated with γ-mangostin and glutamate (Figure 1B). However, when γ-mangostin was treated at 12.5 µM alone, it showed a cell viability of 83.12±3.41%, indicating some cytotoxicity. In addition, it was confirmed that the cell viability that was reduced by glutamate was recovered by the positive control NAC (Figure 1C). As a result, it was found that γ-mangostin significantly inhibited HT22 cell death caused by glutamate at a level similar to that of the positive control, N-acetyl cysteine. Additionally, γ-mangostin turned out to effectively inhibit the modification of HT22 caused by glutamate (Figure 1D).

**Figure 2:**
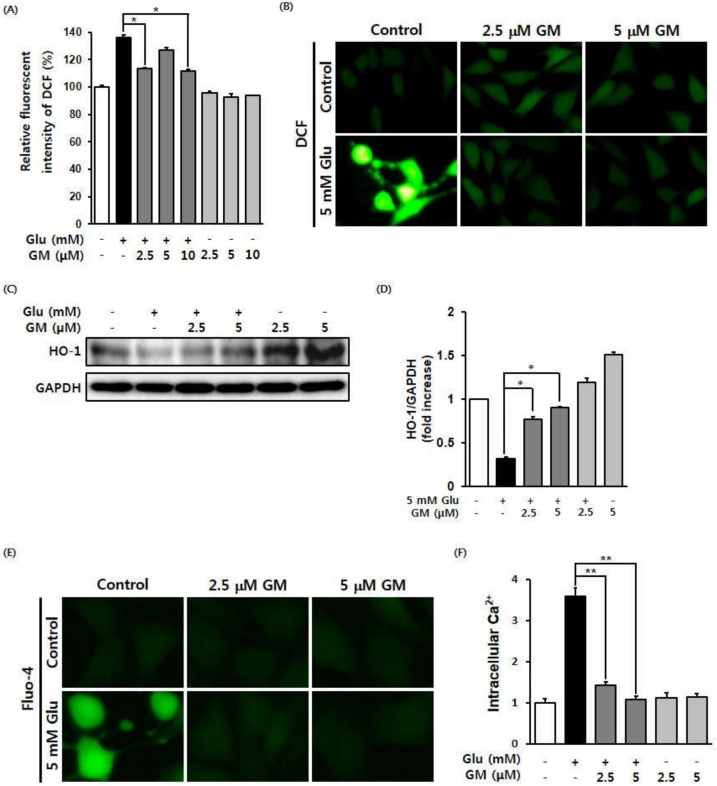
Effect of γ-mangostin (GM) on increased intracellular ROS and Ca^2+^ levels following glutamate (Glu) treatment. (**A**) Inhibitory effect of GM against Glu-induced ROS accumulation in HT22 cells. Cells were treated with 5 mM Glu in the presence or absence of 2.5, 5, and 10 μM GM for 8 h, and the fluorescence intensity of DCF (mean ± standard error of mean [SEM], * *p* < 0.05 compared to Glu-treated cells) was measured. (**B**) Cells were treated with 5 mM Glu along with 2.5 or 5 μM GM for 8 h, and the fluorescence intensity was measured after staining the cells with DCFDA. The images were obtained with a fluorescence microscope (20x magnification). (**C**) Effects of GM on HO-1 protein expression in HT22 cells. Cells were treated with 5 mM Glu along with 2.5 or 5 μM GM for 6 h, and a western blot analysis was performed using HO-1 and GAPDH antibodies. (**D**) Bars denote the fold-increases of HO-1 expression (mean ± SEM. *p < 0.05 compared to Glu-treated cells). (**E**) Inhibitory effect of GM on Glu-induced Ca^2+^ accumulation in HT22 cells. Cells were treated with 5 mM Glu along with 2.5 or 5 μM GM for 8 h and were stained with Fluo-4 AM. The images of cells were obtained using a fluorescence microscope (20× magnification). (**F**) The fluorescence intensity was measured after staining the cells with Fluo-4 AM (mean ± SEM., **p < 0.01 compared to Glu-treated cells).

To determine the effect of glutamate on ROS formation during the cellular damage of HT22 neurons, cells were stained using the fluorescence indicator of ROS, H2DCFDA, with or without γ-mangostin. The accumulation of intracellular ROS was measured by measuring the fluorescence intensity of DCF using a fluorescence plate reader. The results of the DCF fluorescence intensity measurements revealed that the amount of ROS in the glutamate-treated cells increased significantly, whereas cotreatment with γ-mangostin at 2.5 and 10 μM significantly reduced intracellular ROS (Figure 2A). It was also confirmed that the increase in ROS caused by glutamate, as evidenced in the fluorescent images, was significantly reduced by γ-mangostin treatment (Figure 2B). As a protein marker of oxidative stress, HO-1 protein expression was also measured using a western blot analysis. The results confirmed that the reduction in HO-1 protein expression caused by glutamate was restored following γ-mangostin treatment (Figure 2C,D).

Cell permeation of the calcium ion fluorescence indicator, Fluo-4 AM, was examined using a fluorescence microscope. We determined that the fluorescence of the calcium ions had significantly increased when compared to that in the control during oxidative stress caused by the processing of glutamate (Figure 2E). The increased calcium influx by glutamate (3.58%) was decreased to 1.42% and 1.07% by 2.5 and 5 μM γ-mangostin, respectively (Figure 2F).

**Figure 3:**
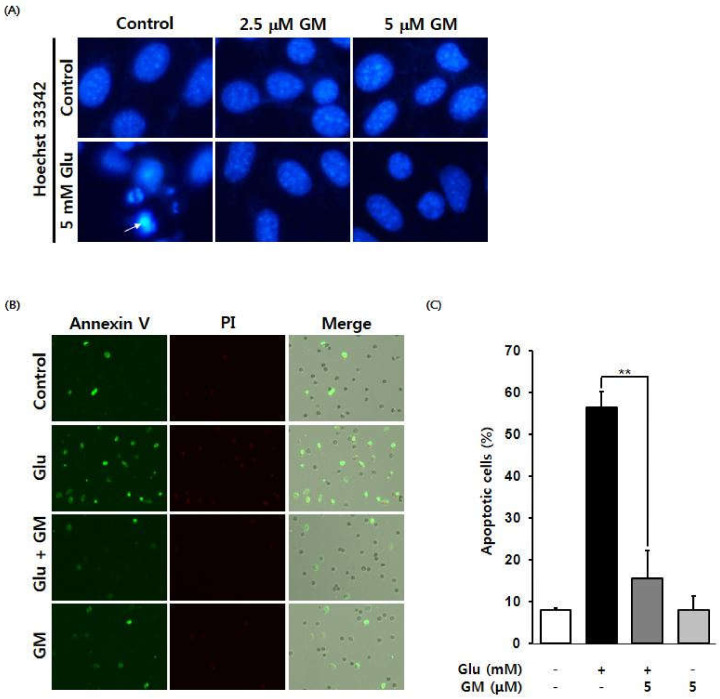
Effect of γ-mangostin (GM) on glutamate (Glu)-induced apoptotic cell death in HT22 cells. (**A**) Inhibitory effect of GM on Glu-induced chromatin condensation in HT22 cells. GM reduced Glu-induced annexin V-positive cells; annexin V-positive cells appear green. (**B**) The effect of GM on apoptosis was measured by fluorescence imaging using a Tali-Image-based cytometer. (**C**) Quantitative representation of the effect of GM on the proportion of apoptotic cells (annexin V-positive cells). Cells were treated with 5 mM Glu and 2.5 or 5 μM GM for 12 h and were then stained with Hoechst 33342. Images were obtained using a fluorescence microscope (20× magnification). Fluorescent images of cells stained using annexin V (green) and PI (red) and a merged image (bottom). The proportion of apoptotic cells was quantitatively analyzed to confirm the effect of GM (mean ± SEM, ** *p* < 0.01 compared to Glu-treated group).

Cells were stained with Hoechst 33,342 to assess the effect of γ-mangostin on chromatin condensation induced by glutamate. The results confirmed that glutamate-induced apoptotic bodies, a result of chromatin condensation, were reduced following treatment with 2.5 and 5 µM γ-mangostin (Figure 3A).

To analyze the induction of apoptosis, HT22 cells were stained with Alexa Fluor 488 and a combination of annexin V and propidium iodide (PI). The proportion of cells with green fluorescence (annexin V-positive cells representing apoptotic cells) increased following glutamate treatment but was significantly reduced when cells were treated with 5 μM γ-mangostin (Figure 3B,C).

**Figure 4:**
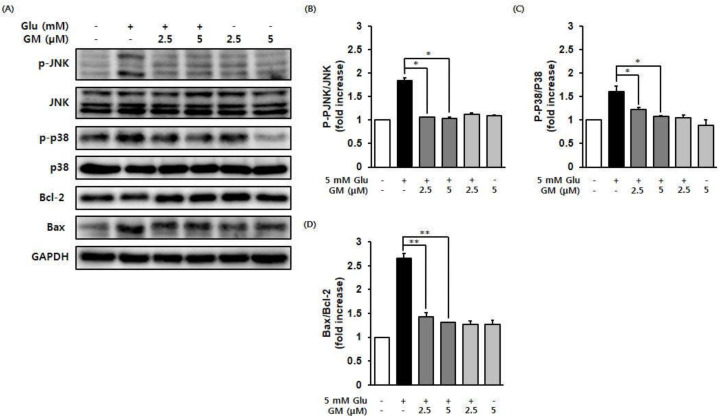
γ-mangostin (GM) inhibits the glutamate (Glu)-induced increase in the phosphorylation of MAPK and the Bax/Bcl-2 ratio. Cells were incubated with 5 mM Glu and 2.5 or 5 μM GM for 6 h. (**A**) The immunoreactive bands were detected with specific antibodies against p-JNK, JNK, p-p38, p38, Bcl-2, Bax, and GAPDH. (**B**,**C**) Bars denote the relative increase of phosphorylation of JNK and p38, respectively (mean ± S.E.M., * *p* < 0.05 and ** *p* < 0.01 compared with glutamate-treated cells). (**D**) The graph represents the ratio of Bax to Bcl-2 protein expression (mean ± S.E.M., ** *p* < 0.01 compared with glutamate-treated cells).

A western blot analysis was performed to determine whether γ-mangostin suppressed the excessive phosphorylation of MAPK induced by glutamate. The results revealed that the phosphorylation of JNK and p38 MAPK, which was increased by glutamate treatment, was effectively suppressed following treatment with γ-mangostin in a concentration-dependent manner (Figure 4). The Bcl-2 family plays a key role in the endogenous apoptotic pathway and controls the membrane permeability of mitochondria. In this study, it was confirmed that the level of the proapoptotic protein, Bax, was increased by glutamate treatment but that γ-mangostin significantly inhibited Bax expression.

## 4. Discussion

If natural antioxidants are used to prevent ROS and neuronal cell death caused by excessive glutamate, active oxygen may be eliminated and homeostasis may be retained, efficiently controlling the mechanism related to cerebropathia such as the decrease of neuronal cell death [19,20,21]. Our study aimed to find out the neuronal cell protective effect and its γ-mangostin mechanism against apoptosis caused by glutamate.

Antioxidants reduce oxidative stress and are closely associated with neuroprotection [22]. It is reported that γ-mangostin is efficient in physiological activities such as anticancer, anti-inflammatory, analgesic and fatty liver disease as well as antioxidative effects [23,24]. Accordingly, when inducing the cell death of the HT22 cell by glutamate in order to verify the brain cell protective effect, γ-mangostin is recovered to the normal state by preprocessing when compared to the control group. NAC acts as an antioxidant after it is converted into cysteine in the body, and removes free radicals as a glutathione precursor that generates glutathione in the liver [25]. It is reported that NAC serves to prevent neurodegenerative diseases, reducing oxidative stress and memory deficits [26,27,28]. It is also found that NAC has protective effects on Cd(NO_3_)_2_-induced cytotoxicity and apoptosis in N2a cells and primary neurons [29]. As a result of using NAC as a positive control in this study, we found that the reported data were consistent with earlier findings. Since glutamate is known to induce cell death by increasing reactive oxygen species in cells, γ-mangostin acts as an antioxidant, efficiently reducing oxidative stress and helping to restore cell viability. Therefore, an ROS inhibition experiment was conducted to determine whether γ-mangostin was effective in reducing oxidative stress. We found that γ-mangostin markedly prevented the accumulation of intracellular ROS induced by glutamate treatment. Treatment of HT22 cells with glutamate increased ROS production as measured by the fluorescence intensity, but the fluorescence intensity was significantly reduced following treatment with γ-mangostin. HO-1 activity protects against oxidative damage both in vitro and in vivo [30]. In a previous study, when natural xanthone components such as α-mangostin, 8-deoxygartanin, gar-tanin, and γ-mangostin were processed at 10 µM, they showed a neuroprotective effect by controlling the antioxidant effect against HT22 cell death, the blood–brain barrier (BBB) and the HO-1 protein level [31]. Therefore, we tested the expression of HO-1, which is sensitive to the induction of oxidative stress. The results showed that HO-1 protein expression was reduced by glutamate. However, treatment with γ-mangostin increased the HO-1 protein expression. In addition, it has been suggested that intracellular Ca^2+^ is a characteristic of neuronal cell death caused by glutamate-induced oxidative stress. Therefore, we also evaluated intracellular Ca^2+^ using Fluo-4 AM, a membrane-permeable fluorescence indicator for Ca^2+^. In this study, the fluorescence intensity decreased when the cells were treated with γ-mangostin. Therefore, the protective effect of γ-mangostin was partly mediated through the inhibition of ROS and Ca^2+^ influx induced by excess glutamate.

Glutamate induces apoptotic cell death in the early stages and necrotic cell death at later stages [18,32]. We first stained HT22 cells with Hoechst 33,342 to observe chromatin condensation, which is a morphological hallmark of apoptotic cell death. We also quantitatively analyzed the proportion of the apoptotic cell population. The result showed that chromatin condensation was detectable in glutamate-treated HT22 cells, whereas γ-mangostin treatment completely prevented the chromatin condensation induced by glutamate. Furthermore, glutamate significantly increased the percentage of annexin V-positive cells, indicative of apoptotic cells, but γ-mangostin significantly reduced the number of apoptotic cells generated following the treatment of HT22 cells with glutamate.

MAPKs are associated with the majority of signal transduction pathways, including those involved in cell differentiation, proliferation, survival, and transformation [33]. JNK and p38 are members of the MAPK family. Several studies have also shown that the regulation of apoptosis involves a host of molecules; in particular, the expression of apoptotic proteins such as Bcl-2 and Bax is altered during the induction of apoptosis [34]. It is reported that γ-mangostin has antioxidant and neuroprotective effects against oxidative stress by controlling the p38 MAPK, Bax/Bcl-2, and caspase-3 activities for toxicity that is induced by 6-OHDA in SH-SY5Y cells [35]. When the effect of α-mangostin and γ-mangostin were compared in cerebral cortex cells, γ-mangostin remarkably inhibited ROS and caspases 3 and 9 activation, and the antiapoptosis activity was superior to α-mangostin [36]. Our results showed that γ-mangostin significantly reduced the phosphorylation of p-JNK and p-p38 proteins induced by glutamate. The regulation of the Bcl-2 and Bax mechanisms was also confirmed.

## 5. Conclusions

Several studies have suggested the role of oxidative stress-induced damage in many neurodegenerative diseases [37]. In addition, there is a growing interest in the protective effect of natural products against oxidative neuronal damage [38,39]. In this study, we demonstrate that γ-mangostin is a bioactive compound that offers significant neuroprotective benefits. To the best of our knowledge, this is the first report to show that γ-mangostin, a radical scavenger, inhibits the production of ROS, apoptosis, and the expression of the JNK and p38 signaling pathways, thus protecting HT22 cells against glutamate-induced cytotoxicity. However, further studies are needed to identify the precise mechanisms of regulation by γ-mangostin and to verify these effects in animal models of neurological diseases.

## Data Availability

The data presented in this study are available on request from the corresponding author.

## Supplementary Materials

The following are available online at https://www.mdpi.com/2218-273X/11/2/170/s1, Figure S1: ^1^H NMR spectrum of γ-mangostin, Figure S2: ^13^C NMR spectrum of γ-mangostin, Figure S3: UPLC UV chromatogram of γ -mangostin, Figure S4: MASS spectral data of γ-mangostin.
