# Supplementary material for: Protective Effect of γ-mangostin Isolated from the Peel of Garcinia mangostana against Glutamate-Induced Cytotoxicity in HT22 Hippocampal Neuronal Cells
Source: Biomolecules. 2021 Jan 27;11(2):170. doi: 10.3390/biom11020170 (PMC7910862; doi:10.3390/biom11020170)
Supplement: Supplementary file 1 [file biomolecules-11-00170-s001.pdf]

Supporting Information

Protective effect of γ-mangostin isolated from the peel of *Garcinia mangostana* against glutamate-induced cytotoxicity in HT22 hippocampal neuronal cells

Ji-Yun Baek[1,2,†], Kiwon Jung[3,†], Young-Mi Kim[4], Hyun-Young Kim[2,*], Ki Sung Kang[1,*] and Young Won Chin[4,*]

Contents

Figure S1. The ^{1}H NMR spectrum of γ-mangostin (CD_3OD, 400 MHz)

Figure S2. The ^{13}C NMR spectrum of γ-mangostin (CD_3OD, 100 MHz)

Figure S3. The UPLC UV chromatogram of γ-mangostin (detected at 320 nm)

Figure S4. The MASS spectral data of γ-mangostin

Extraction and isolation

The dried peel of *Garcinia mangostanta* L (1.23kg) was extracted with EtOH three times. The EtOH extract (87.1g) was suspended in water and then partitioned with $CHCl_3$, EtOAc and *n*-BuOH, yielding 50.18g, 2.74g, and 18.27g, respectively. The $CHCl_3$ fraction (45.93g) was chromatographed over a silica gel column chromatography using gradient with $CHCl_3$- MeOH (100:0 to 0:100) as solvent and obtained 18 subfraction (GMC1-GMC18). GMC12 (4.73 g) fraction was separated using preparative RP-MPLC with gradient mixture of 40% to 80% MeOH to yield γ-mangostin (3.20 g). The structure of γ-mangostin was confirmed by comparing the measured NMR data with the published values [1,2].

γ-mangostin: HRESIMS*: m/z* 397.1652 $[M+H]^+$(calcd for $C_{23}H_{25}O_6$, 397.1651) ^{1}H NMR (CD_3OD, 400 MHz) δ 6.67 (1H, s, H-5), 6.24 (1H, s, H-4), 5.25 (2H, m, H-2′, 2″), 4.12 (2H, d, *J* = 5.9 Hz, H-1″), 3.34 (2H, m, H-1′), 1.84 (3H, s, H-4″), 1.78 (3H, s, H-4′), 1.66 (6H, s, H-5′, 5″); ^{13}CNMR (CD_3OD, 100 MHz) δ 183.8 (C-9), 163.5 (C-3), 161.7 (C-1), 156.5 (C-6), 154.2 (C-4a), 153.4 (C-10a), 142.2 (C-7), 131.9 (C-3′), 131.8 (C-3″), 129.8 (C-8), 125.0 (C-2″), 124.1 (C-2′), 112.4 (C-2), 111.3 (C-8a), 104.1 (C-9a), 101.1 (C-5), 93.1 (C-4), 26.8 (C-1″), 26.2 (C-5″), 26.1 (C-5′), 22.4 (C-1'), 18.5 (C-4′), 18.1 (C-4″).

UPLC-UV analysis for the purity of γ-mangostin

Chromatographic analysis for the purity of γ-mangostin was performed on an Acquity UPLC system with an ACQUITY UPLC®BEH C18 column (2.1 ×150 mm, 1.7 μm, Waters, Milford, MA, USA). An aliquot (3 μL) of the sample (0.1mg/mL in MeOH) was injected into the UPLC system. The mobile phase consisted of 0.1% formic acid in water (A) and 0.1% formic acid in acetonitrile (B). The mobile phase consisting of (A) and (B) was run at a flow rate of 0.3 mL/min by the following programmed gradient elution: 40% (B, v/v) isocratic for 15min, 40→100% (B) in 5min, 100% (B) isocratic for 3min, 100→40% (B) in 0.5min, 40% (B) isocratic for 1.5min as post-run for reconditioning. The column temperature was maintained at 40 ℃ and the wavelength was set at 320 nm

Reference

1. Xu, Zeng; Huang, Lei; Chen, Xiao-Hong; Zhu, Xiao-Feng; Qian, Xiao-Jun; Feng, Gong-Kan; Lan, Wen-Jian; Li, Hou-Jin. Cytotoxic prenylated xanthones from the prericarps of *Garcinia mangostana*. *Molecules*, 2014, 19, 2, 1820-1827.

2. Chin, Xiao-Qian; Zi, Cheng-Ting; Li, Hong-Mei; Yang, Liu; Lv, Yong-Feng; Li, Jin-Yu; Hpu, Bo; Ren, Fu-Cai; Hu, Jiang-Miao; Zhou, Jun. Design, synthesis and structure-activity relationships of mangostin analogs as cytotoxic agents. *RSC Advances*, 2018, 8, 72, 41377-41388.

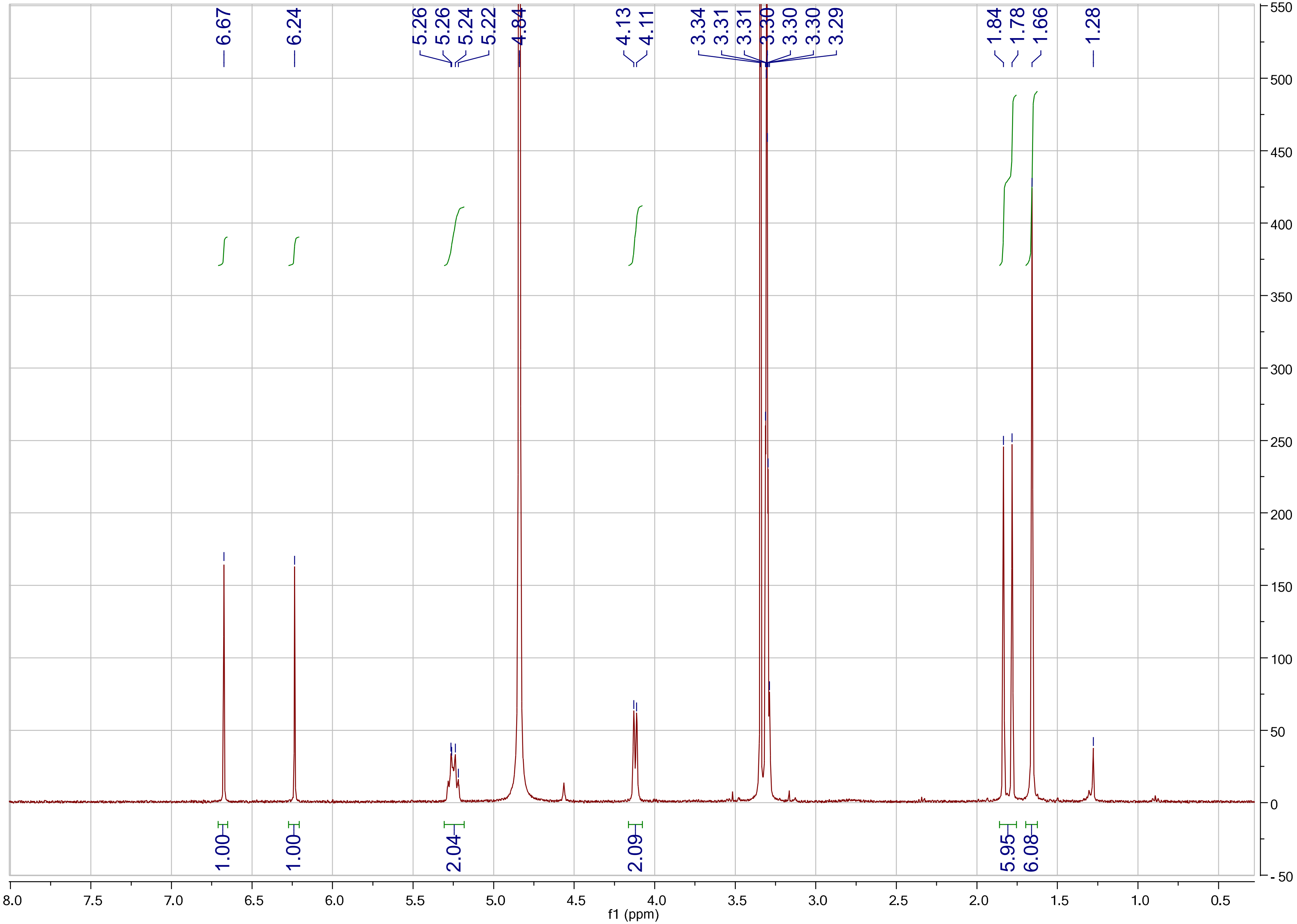

Figure S1. ^{1}H NMR spectrum of γ-mangostin in (CD_3OD, 400 MHz)

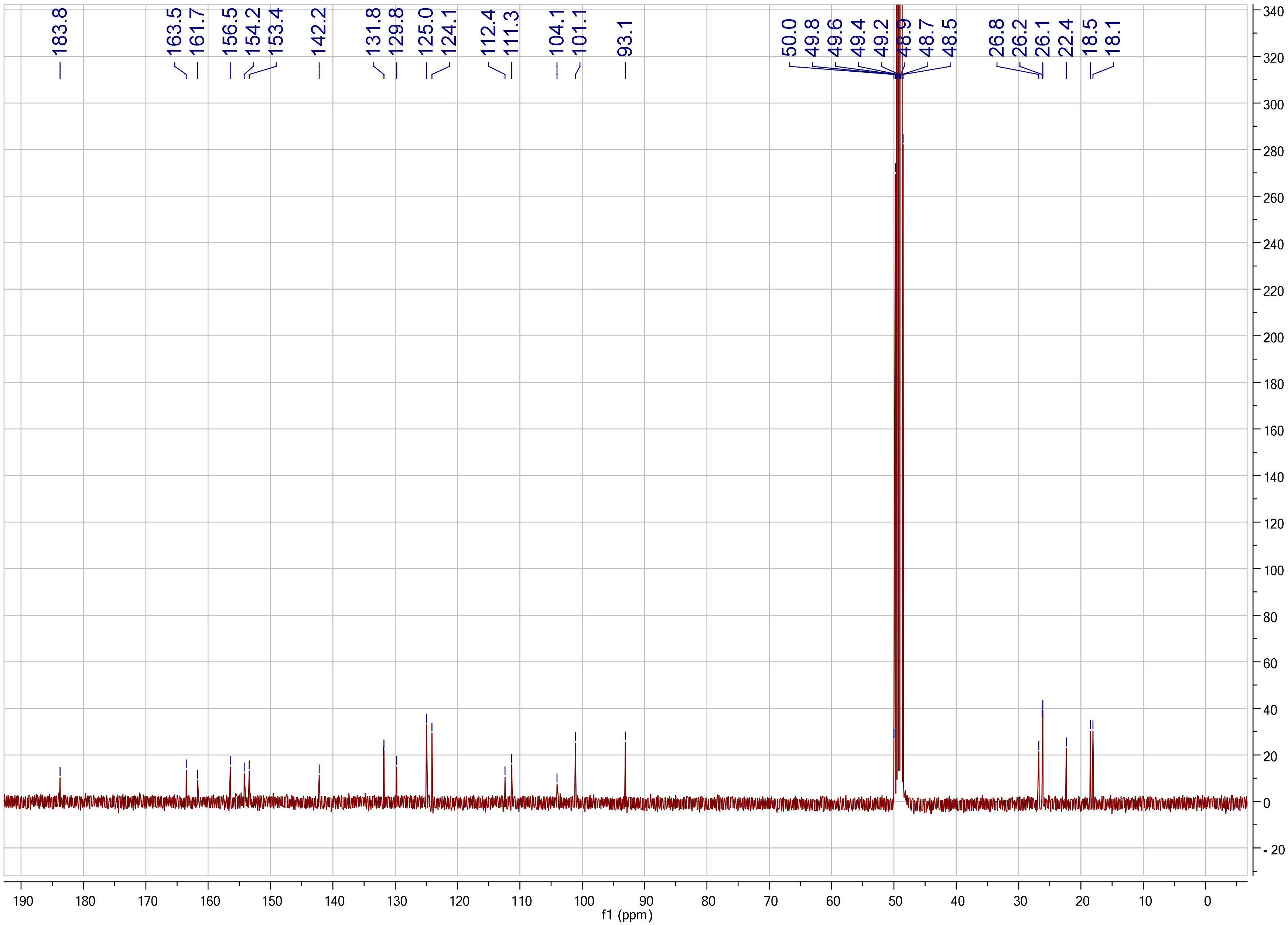

Figure S2. ^{13}C **NMR spectrum of γ -mangostin (CD$_3$OD, 100 MHz)**

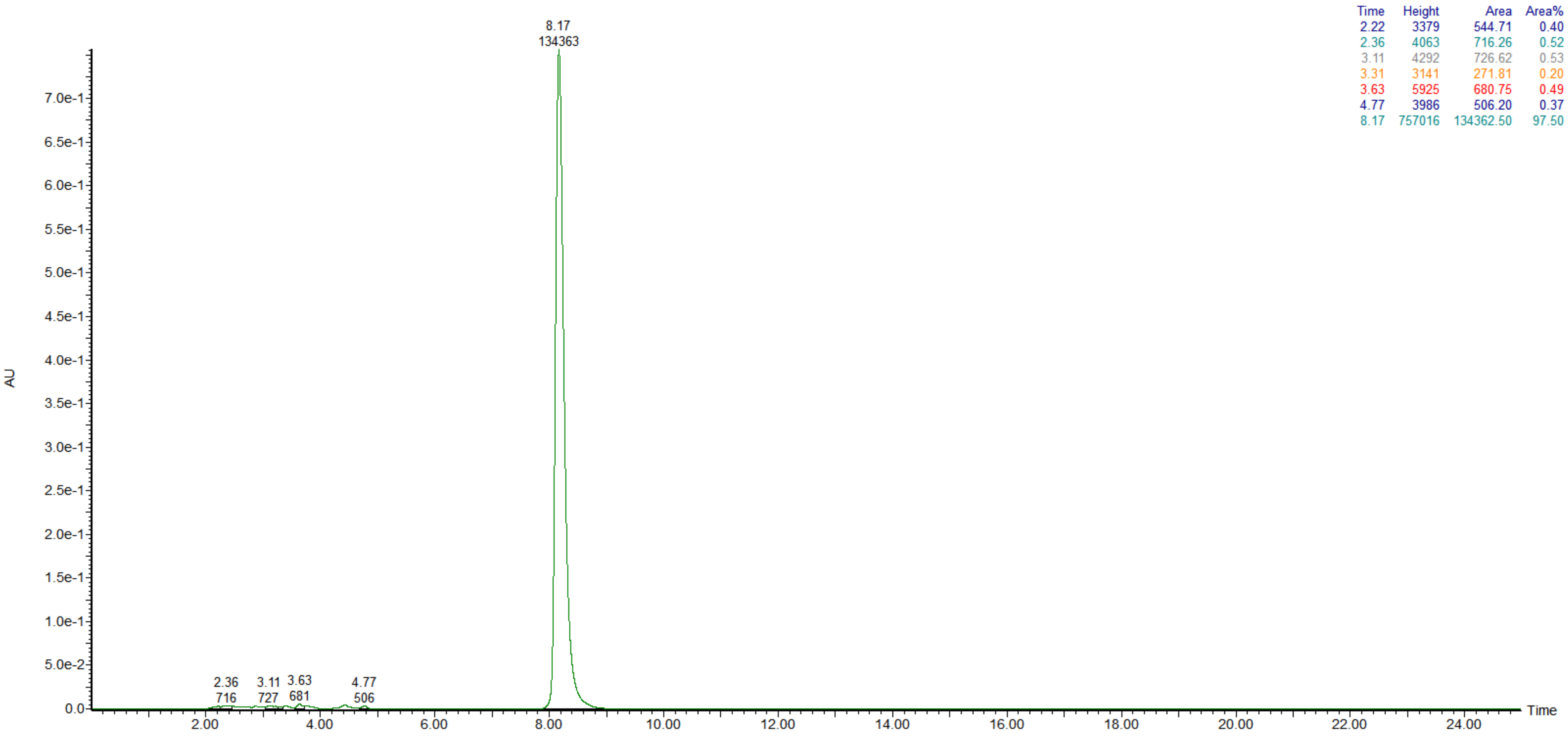

Figure S3. The UPLC UV chromatogram of γ -mangostin (UV wavelength 320nm)

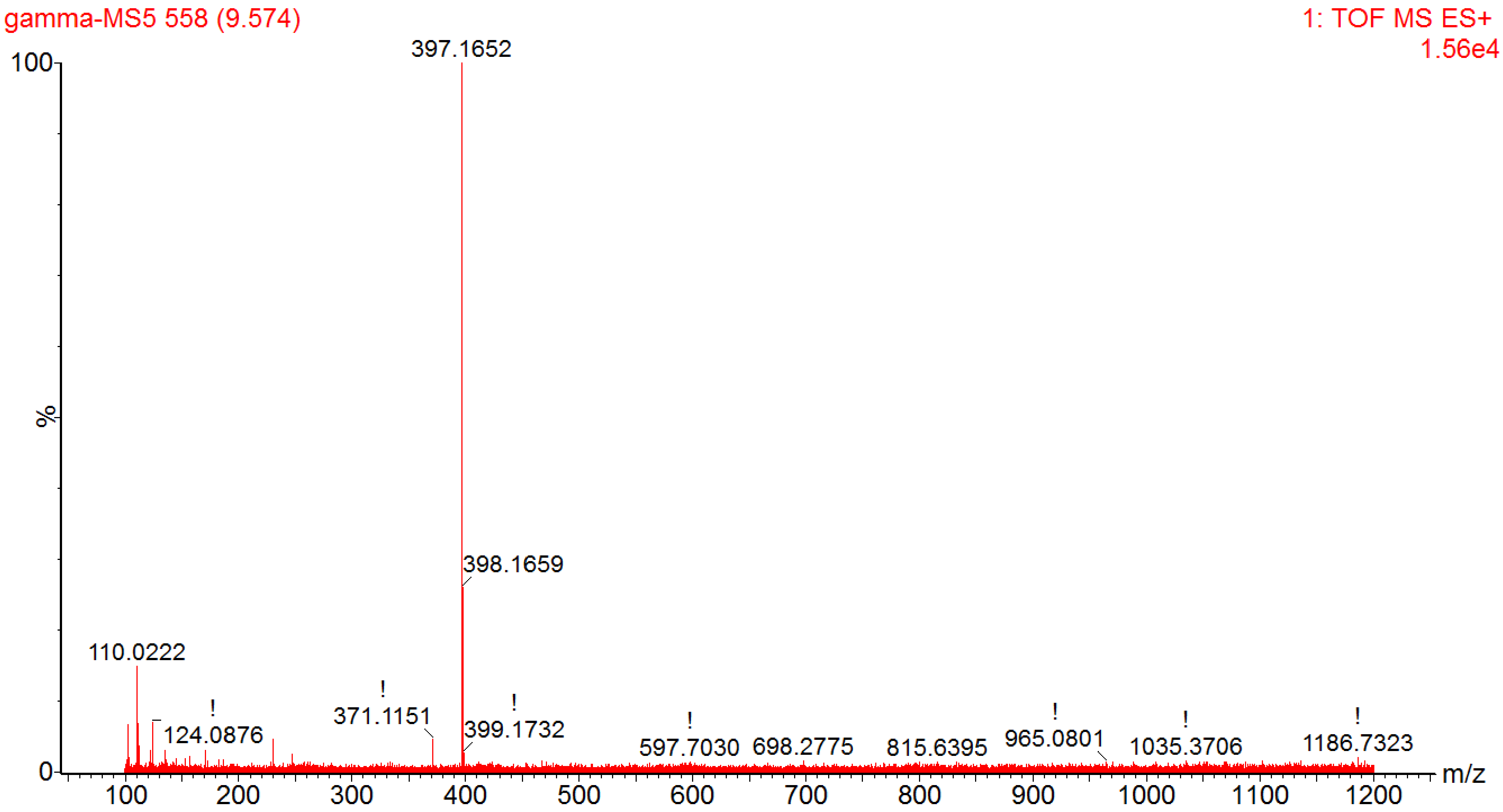

Figure S4. The MASS spectral data of γ-mangostin
